# Primary cardiac angiosarcoma resection and reconstruction with pedicled autologous pericardium: A case report

**DOI:** 10.1016/j.ijscr.2020.03.045

**Published:** 2020-04-03

**Authors:** Hirotake Gonda, Masato Nakayama, Masashi Toyama, Takehito Kato

**Affiliations:** a Department of General Surgery, Toyohashi Municipal Hospital, Aichi, Japan; b Department of Cardiovascular Surgery, Toyohashi Municipal Hospital, Aichi, Japan

**Keywords:** Cardiac angiosarcoma, Pedicled autologous pericardium, Reconstruction

## Abstract

**Introduction:**

Primary cardiac angiosarcoma is rare, and its prognosis remains poor. Complete surgical resection is the first choice among the available treatments, to ensure prolonged patient survival.

**Presentation of case:**

We report the case of a 72-year-old man with right atrial angiosarcoma who presented with bloody pericardial effusion due to tumor hemorrhaging. Emergency surgery was performed. The right atrial free wall and vena cava were reconstructed with pedicled autologous pericardium. The patient died 12 months later because of multiple organ failure due to metastasis. Autopsy showed a maintained right atrial chamber and only mild calcification.

**Discussion:**

Pedicled autologous pericardium may have contributed to preserving cardiac function that could withstand chemotherapy and radiotherapy.

**Conclusion:**

Pedicled autologous pericardium was found to be useful for reconstructing the cardiac chamber after removal of a cardiac tumor.

## Highlights

• This article is about a 72-year-old man with right atrial angiosarcoma.
• We performed reconstruction using pedicled autologous pericardium.
• Pedicled autologous pericardium was found to be useful for reconstructing.

## 1 Introduction

The prognosis of primary cardiac angiosarcoma is poor [1]. Complete surgical resection is the first choice for prolonged patient survival [1]. Free autologous pericardium or xenopericardium is used for cardiac reconstruction in most cases [2,3]. We present a case of primary cardiac angiosarcoma, in which the right atrium (RA) and vena cava were reconstructed using pedicled autologous pericardium. We discuss the potential benefits of pedicled autologous pericardium for cardiac reconstruction after primary cardiac tumor resection. This work has been reported in line with the SCARE criteria [4].

## 2 Case presentation

A 72-year-old man was referred to our hospital for a 10-day history of systemic edema. On admission, his blood pressure was 159/112 mmHg, heart rate was 97/min, and respiratory rate was 27/min. Echocardiography revealed a massive pericardial effusion. Pericardiocentesis yielded bloody pericardial fluid. An enhanced computed tomography (CT) scan revealed an irregular mass located in the RA wall, and bilateral pulmonary nodules.

**Fig. 1:**
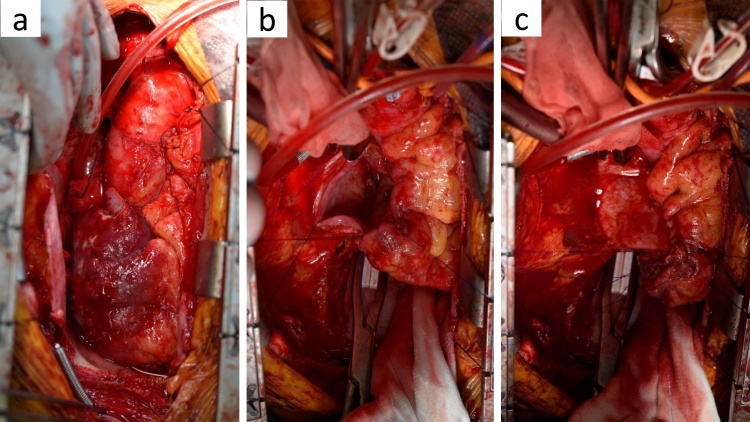
a. The hemorrhaging tumor was located in the RA, SVC, and IVC. b. After resecting the tumor. c. Reconstruction with pedicled autologous pericardium.

Emergency surgery was performed because the tumor was hemorrhaging into the pericardial cavity. To enable extensive resection, a cardiopulmonary bypass with aortic return and venous drainage via the femoral vein and extra-pericardial superior vena cava was started outside the pericardial cavity. The irregular and hemorrhaging tumor was located in the RA, superior vena cava (SVC), and inferior vena cava (IVC) (Fig. 1a). The RA free wall and proximal parts of SVC and IVC were excised (Fig. 1b). All tumors were excised macroscopically. After mobilization of the right-sided pericardium, we sutured it to the resected part in a pedicled manner using running polypropylene suture (Fig. 1c).

**Fig. 2:**
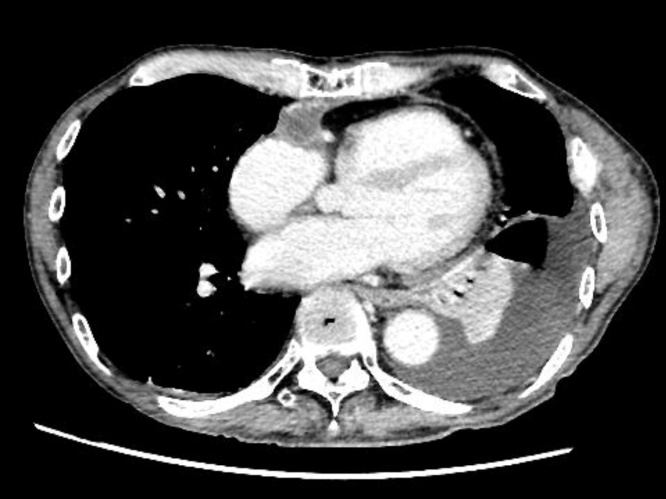
Enhanced computed tomographic scan showed the right atrial wall lumen was not narrowed. Pleural and pericardial effusion are observed.

The pathological diagnosis was angiosarcoma. On the 2th postoperative day, he was extubated. On the 5th postoperative day, he resumed diet. On the 19th postoperative day, positron emission tomography showed local recurrence and spinal metastasis. Therefore, he started radiation therapy for each lesion. On the 39th postoperative day, chemotherapy with paclitaxel (80 mg/m^2^ once every 3 weeks) was initiated. He was discharged on the 75th postoperative day. During the follow-up period, he showed no symptoms of right heart failure. Echocardiography and enhanced CT scan showed a preserved reconstruction lumen (Fig. 2). Despite chemotherapy, CT showed marked that lung and liver metastases had clearly worsened at 11 months after surgery. He died of multiple organ failure due to metastasis 12 months after surgery. On autopsy, the RA cavity and vena cava were not narrowed, no blood clot was seen, and pericardial calcification was mild.

## 3 Discussion

We used pedicled autologous pericardium for cardiac reconstruction after removing the heart tumor. The results suggest that pedicled autologous pericardium was useful for maintaining cardiac function after reconstruction. Pedicled autologous pericardium has been used for various reconstructive procedures in congenital cardiovascular surgery [5] and free autologous pericardium or xenopericardium have been commonly used to reconstruct cardiac defects after cardiac tumor removal [2,3]. Adachi et al. [5] reported that pedicled autologous pericardium, which was used as a tube graft in a primary Fontan procedure, was pliable and non-calcified upon reoperation 9 years later. They also stated that the pedicled autologous pericardium might differentiate to resemble the vascular wall tissue after having been used as a vascular substitute. Masaki et al. [6] found that pedicled autologous pericardium tissue microvasculature was clearly preserved compared to the free pericardium implanted for right ventricular outflow reconstruction, and suggested its good long-term function and durability. In our case, as in the studies by Adachi et al. [5] and Masaki et al. [6], the reconstructed part showed only mild calcification and no thrombus or narrowing. These features may have contributed to preserving cardiac function that could withstand chemotherapy and radiotherapy. As the tumor and pericardium are close to each other, reconstruction using autologous pericardium raises concerns of local recurrence. In our case, local recurrence occurred, but it was controlled with radiotherapy and chemotherapy.

## 4 Conclusion

Primary cardiac angiosarcoma currently has a poor prognosis. However, if long-term survival can be expected with novel adjuvant therapy in the future, postoperative cardiac function is important. Pedicled autologous pericardium was found to be useful for reconstructing the cardiac chamber after removal of a cardiac tumor, especially regarding better postoperative cardiac function.

## Declaration of Competing Interest

The authors have no conflicts of interest.

## Funding

None.

## Ethical approval

Case reports are exempt from ethnical approval in our institution.

## Consent

The patient’s family members provided informed consent for this study to be published. A copy of the written consent is available for review by the Editor-in-Chief of this journal on request.

## Author contribution

Hirotake Gonda wrote the manuscript. Masato Nakayama, Masashi Toyama and Takehito Kato supervised the writing of the manuscript.

## Registration of research studies

Not applicable for a case report.

## Guarantor

Hirotake Gonda.

## Provenance and peer review

Not commissioned, externally peer-reviewed.

## References

[1] Patel S.D., Peterson A., Bartczak A., Lee S., Chojnowski S., Gajewski P. (2014). Primary cardiac angiosarcoma – a review. Med. Sci. Monit..

[2] Benassi F., Maiorana A., Melandri F., Stefanelli G. (2010). A case of primary cardiac angiosarcoma: extensive right atrial wall reconstruction with autologous pericardium. J. Card. Surg..

[3] Abu Saleh W.K., AI Jabbari O., Ramlawi B., Bruckner B.A., Loebe M., Reardon M.J. (2016). Right atrial tumor resection and reconstruction with use of an acellular porcine bladder membrane. Tex. Heart Inst. J..

[4] Agha R.A., Borrelli M.R., Farwana R., Koshy K., Fowler A., Orgill D.P., For the SCARE Group (2018). The SCARE 2018 statement: updating consensus Surgical CAse REport (SCARE) guidelines. Int. J. Surg..

[5] Adachi I., Yagihara T., Ishibashi-Ueda H., Kitamura S. (2006). Immunohistological findings for an extracardiac conduit in Fontan pathway constructed with pedicled autologous pericardium. Eur. J. Cardiothorac. Surg..

[6] Masaki N., Adachi O., Kawamoto S., Saiki Y. (2017). Implanted pedicled autologous pericardium mimics vasculature tissue: case report. J. Thorac. Cardiovasc. Surg..

